# Fungal-bacterial interactions and their relevance to oral health: linking the clinic and the bench

**DOI:** 10.3389/fcimb.2014.00101

**Published:** 2014-07-29

**Authors:** Patricia I. Diaz, Linda D. Strausbaugh, Anna Dongari-Bagtzoglou

**Affiliations:** ^1^Division of Periodontology, Department of Oral Health and Diagnostic Sciences, The University of Connecticut Health CenterFarmington, CT, USA; ^2^Department of Molecular and Cell Biology, The Center for Applied Genetics and Technologies, The University of ConnecticutStorrs, CT, USA

**Keywords:** oral health, microbiome, mycobiome, fungi, bacteria, interactions

## Abstract

High throughput sequencing has accelerated knowledge on the oral microbiome. While the bacterial component of oral communities has been extensively characterized, the role of the fungal microbiota in the oral cavity is largely unknown. Interactions among fungi and bacteria are likely to influence oral health as exemplified by the synergistic relationship between *Candida albicans* and oral streptococci. In this perspective, we discuss the current state of the field of fungal-bacterial interactions in the context of the oral cavity. We highlight the need to conduct longitudinal clinical studies to simultaneously characterize the bacterial and fungal components of the human oral microbiome in health and during disease progression. Such studies need to be coupled with investigations using disease-relevant models to mechanistically test the associations observed in humans and eventually identify fungal-bacterial interactions that could serve as preventive or therapeutic targets for oral diseases.

## Introduction

Around 600 bacterial species and a still undetermined number of fungal species inhabit the oral cavity of humans (Dewhirst et al., 2010; Ghannoum et al., 2010; Dupuy et al., 2014). Oral microbial cells arrange in organized biofilm structures on non-shedding surfaces such as teeth. Organized aggregates are also formed on mucosal surfaces and even in the salivary fluid phase, via specific cell to cell adhesion events (Dongari-Bagtzoglou et al., 2009; Kolenbrander et al., 2010). Such physical proximity facilitates metabolic interactions among microbial cells (Egland et al., 2004; Jakubovics et al., 2008; Kim et al., 2008), while a defined spatial structure has been shown to increase stability of microbial communities, allowing creation of chemical gradients (Kim et al., 2008). Microorganisms in these diverse assemblages interact through various types of metabolic exchanges. For example, bacterial consortia cooperate to release nutrients from macromolecules available in oral fluids (Bradshaw et al., 1994). Cross-feeding events have been identified in which metabolic end-products of one species are used as carbon sources by another community member (Diaz et al., 2002; Marsh and Martin, 2009). Also, through various signaling events, bacterial cells coexisting in a community alter the phenotype of their neighbors (Kuboniwa et al., 2009; Frias-Lopez and Duran-Pinedo, 2012). Due to their specific nature, microbial interactions are likely to influence community assembly and may also determine resistance and resilience of communities to perturbations. Since most oral diseases are associated with perturbations of community balance, understanding the interactions among species that maintain community stability or allow microbial shifts to occur is an essential part of the development of strategies to preserve and restore oral health. Interactions among oral microbial cells, however, have almost been exclusively studied in bacteria, while little is known regarding fungi-bacteria relationships.

**Figure 1:**
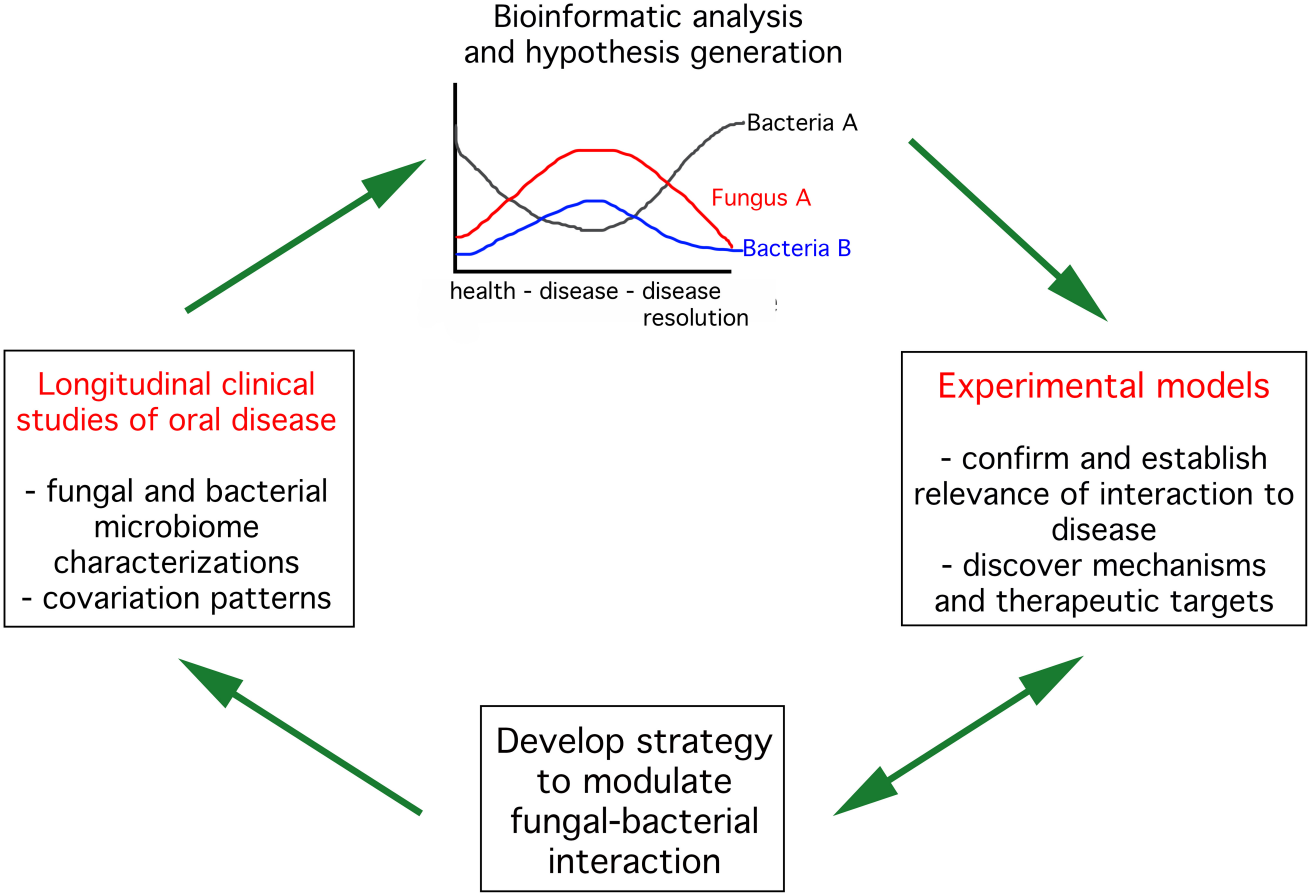
**Strategies to advance knowledge on fungal bacterial interactions in the context of oral health**. The first step (left panel) is to conduct longitudinal clinical studies in humans characterizing the fungal and bacterial components of the microbiome throughout progression and resolution of oral diseases. These observational studies should lead to identification of potential positive (top panel, fungus A and bacteria B) or negative (top panel, fungus A and bacteria A) interactions between bacteria and fungi. Selected interactions need to be tested in disease-relevant experimental models to confirm their importance to disease progression and dissect mechanisms mediating the interaction. These studies could then be used to develop strategies to interfere with fungal-bacterial interactions. Such strategies need to be tested in experimental model systems and eventually in humans.

Limited interest on fungal-bacterial interactions and their role in health and disease is a consequence of incomplete knowledge on the fungal microbiota. Most studies on oral fungi have focused on *Candida* species, which are highly amenable to cultivation. *Candida* species establish in the oral cavity as commensals but may become virulent, causing mucosal lesions, under certain conditions (Lalla et al., 2013). The role oral bacteria play in candidiasis has only recently received attention (Diaz et al., 2012b; Xu et al., 2013). Interest in fungi other than *Candida* and their role in oral health and disease is also emerging as recent molecular characterizations of the oral mycobiome have highlighted the great diversity of fungi present in the oral cavity (Ghannoum et al., 2010; Dupuy et al., 2014). In this perspective we will summarize current understanding of fungal-bacterial ecology in relation to oral health, arguing that while the advent of “omics” approaches will facilitate the identification of potential fungal-bacterial relationships associated with health and disease, parallel mechanistic studies using *in vitro* and *in vivo* models are needed. Thus, a link between clinical studies in humans conducted via a systems biology perspective and laboratory experimental approaches using models to study candidate fungal-bacterial relationships will provide the key to understand the roles of fungal-bacterial interactions in oral health maintenance (Figure 1).

## “Omics” technologies revolutionized characterization of bacterial and fungal oral communities

A variety of “omics” approaches, particularly those powered by high throughput DNA sequencing accelerated knowledge on the composition of oral microbial communities. Parallel sequencing of 16S rRNA-based amplicon libraries allows rapid identification of bacterial species present in oral samples in what has become a relatively straight forward process (Diaz et al., 2012a). The availability of curated 16S rRNA sequence databases for oral taxa, such as the Human Oral Microbiome Database (HOMD), facilitates assignment of species level taxonomies to short sequence reads (Dewhirst et al., 2010). We use the 16S rRNA hypervariable regions V1 and V2 as a tool to survey the oral bacteriome. Using this 16S rRNA region, it is possible to obtain species level taxonomic identities with almost the same accuracy as when using full length 16S rRNA gene sequences. For instance, if we download the 830 full length 16S rRNA sequences from the HOMD and classify each sequence using Mothur's version of the Ribosomal Database Project classifier, with a bootstrapping cutoff of 80% (Schloss and Westcott, 2011), and using the same HOMD as template, we are able to correctly assign ~92% of sequences to their respective species. Those sequences for which a species level taxonomy is not possible belong to species undistinguishable by their 16S rRNA sequences (e.g., *Streptococcus mitis* and *Streptococcus oralis*) or to organisms for which taxonomies still need clarification (e.g., *Peptosptreptococcacceae, Alloprevotella* spp.). When using only the V1-V2 regions, we are able to correctly assign ~90% of HOMD sequences to species, in close agreement to results using full length 16S rRNA. It is thus possible to perform accurate species-level taxonomic surveys of oral communities using partial V1-V2 16S rRNA amplicons.

Using high throughput sequencing of 16S rRNA gene fragments, our group and others have defined the complex shifts in subgingival bacterial communities associated with periodontitis, an inflammatory condition of the supporting structures of teeth (Griffen et al., 2011; Abusleme et al., 2013). These studies have shown that despite great inter-subject variability in microbiome composition, dozens of species are consistently associated with periodontitis. Similarly, the bacterial microbiomes associated with caries have been characterized revealing a complex community in which the acidogenic microorganism *Streptococcus mutans* becomes abundant in most, but not all, caries-associated microbiomes (Gross et al., 2012). Moreover, high throughput sequencing is being used to evaluate community functions in health and disease, by comparison of shifts in the metagenomes and metatranscriptomes of plaque samples (Belda-Ferre et al., 2012; Duran-Pinedo et al., 2014; Jorth et al., 2014). These studies, however, have targeted only the bacterial component of the microbiome.

In contrast to the oral bacterial microbiota, knowledge on the fungal microbiota is limited. Our group has used high throughput sequencing of internal transcribed spacer 1 (ITS1) amplicon libraries to characterize the fungi present in oral samples. As with bacterial amplicons, the actual process of sequencing is relatively clear-cut, but there are three special challenges associated with the mycobiome (Dupuy et al., 2014). First, some fungal cells are notoriously difficult to break open, so we have adopted a relatively harsh bead-beating method that utilizes a very high density zirconia bead. We suspect this improvement allowed us to be the first to capture the widespread and abundant presence of species from the genus *Malassezia* in the healthy human mouth. A second challenge is to improve the legitimacy and accuracy of taxonomic assignments. Using the curated Fungal Metagenomics Project database to analyze our sequence datasets, we have empirically developed a BLAST E-value match statistic (10^−42^) that reduces spurious assignments and improves the likelihood of identification of biologically relevant fungi. The third challenge, and one likely to be of considerable significance to understanding fungal involvement in oral health and disease, are the fungal-specific problems in binary names and phylogenetic classifications. Fungi provide the most abundant and widespread examples of organisms with multiple names, often involving different genera, lacking nomenclature guidelines. Three factors contribute to autonomous naming: independent isolation of the same fungus from different environments, dimorphic forms, and the presence of asexual (anamorph) and sexual (teleomorph) pairs. With respect to the oral cavity, the anamorph and teleomorph pairs of many *Candida* and *Pichia* species provide instructive examples. In our studies, *Cyberlindnera jadinii* (from NCBI) has been exclusively represented by its synonym, *Pichia jadinii*, which has the anamorph name of *Candida utilis*; in fact, anamorphic genus *Candida* names exist for each of the teleomorphic *Pichia* species we have found in the mouth to date. In the example mentioned, we have decided to use *Candida* as the priority genus referring to these sequences as *Candida utilis* (*Pichia jadinii, Cyberlindnera jadinii*). There is, however, an urgent need to develop a curated database for oral fungi with consensus nomenclature to link previous and current clinical studies.

Using the aforementioned improvements, we have examined the salivary mycobiome in healthy individuals via ITS1 sequencing (Dupuy et al., 2014). Our results are in good agreement with the only similar study (Ghannoum et al., 2010) in identifying consensus mycobiome members *Candida/Pichia, Cladosporium/Davidiella, Alternaria/Lewia, Aspergillus/Emericella/Eurotium, Fusarium/Gibberella, Cryptococcus/Filobasidiella*, and *Aureobasidium*. Our study, however, was the first to identify *Malassezia* species as prominent commensals. We have now extended our analysis to dozens of samples from healthy individuals that were collected in different clinical or research environments, and have confirmed widespread presence of *Malassezia* species in the mouth. The role of *Malassezia* spp. in oral homeostasis, however, remains unknown.

## Using “omics” information to identify candidate fungal-bacterial interactions important to oral health

Microbiome profiles can be used to explore co-occurrence and co-exclusion patterns in oral communities. Associations inferred from this analysis could be used to generate hypotheses regarding synergistic or antagonistic interactions among fungi and bacteria. Several approaches to this analysis have been proposed. Duran-Pinedo et al. (2011) used weighted correlation network analysis to identify associations between bacterial species in subgingival plaque. In a proof of principle experiment, these authors demonstrated that correlation network information could improve the growth of uncultivated taxa in laboratory media. Several *Prevotella* spp. were identified as candidate growth partners for the uncultivated *Tannerella* sp. HOT286, based on direct associations in microbial network modules. Using the helper *Prevotella* spp. in a co-cultivation approach, the authors were able to enrich for *Tannerella* sp. HOT286 in solid laboratory media.

More recently, Faust et al. analyzed the 16S rRNA-based microbial profiles of more than 5000 samples from healthy individuals to infer a bacterial interaction network, addressing the methodological limitations of using simple correlation coefficients such as Pearson's or Spearman's to analyze organismal associations from relative abundance data (Faust et al., 2012). Since relative abundance measures are dependent on each other and the increase in one organism is always accompanied by a decrease in others, these authors devised a series of analytical approaches to account for the compositionality of the data, inferring significant inter-species associations at different body sites.

The only publication to date that has used microbial 16S rRNA and ITS profiles to explore co-occurrence patterns among fungal and bacterial members of oral communities is that by Mukherjee et al. (2014). These authors report on a series of pairwise Spearman's correlation tests for bacterial and fungal genera present in oral rinse samples and then explore an antagonistic relationship between two oral fungi, *Candida* and *Pichia*. Validation of the fungal-bacterial correlations in larger cohorts and experimental evidence demonstrating their significance are still required.

Amplicon-based profiles have also been correlated to total and taxon-specific loads in oral samples. Kraneveld et al. combined 16S rRNA gene profiling with real time qPCR measurements to explore the relationship between *Candida* load, bacterial load and the bacterial microbiome composition of saliva in elderly subjects (Kraneveld et al., 2012). After comparison of the 16S rRNA to *Candida* ITS qPCR ratios, it was seen that in most subjects bacteria outnumbered *Candida*. However, in one subject *Candida* appeared at much higher levels than bacteria. Interestingly, the authors found that in samples with high *Candida* load, there was an increase in relative abundance of saccharolytic species from the genera *Streptococcus, Lactobacillus*, and *Scardovia*, among others, suggesting a relationship between an acidogenic flora and *Candida*.

No human clinical study to date has investigated associations among bacteria and fungi in a longitudinal manner simultaneously evaluating disease progression or therapy outcomes. Such longitudinal and/or interventional studies could reveal whether oral diseases are associated with disruption of fungal-bacterial relationships. For instance, the incidence of candidiasis in non-oral mucosal compartments such as the vaginal tract is associated with antibiotic intake and it is therefore believed to be a consequence of disrupting the bacterial microbiome (Maraki et al., 2003; Xu et al., 2008). Indeed, evidence from animal models suggests that in the gut *Candida* interacts with the resident bacterial microbiome potentially influencing host-microbiome homeostasis (Mason et al., 2012a,b). Using a rodent model exposed to a cephalosporin antibiotic, Mason et al. (2012a,b) demonstrated that an intact bacterial flora is essential to prevent *Candida* colonization of the lower gastrointestinal tract. In turn, *C. albicans* colonization of microbiome-perturbed mice promoted sustained gut dysbiosis, preventing the regrowth of lactobacilli, which are presumably associated with gastrointestinal health, while allowing establishment of *Enterococcus* spp. at higher levels than those present prior to antibiotic treatment. Moreover, *in vitro* evidence suggests that *C. albicans* virulence may be modulated by the bacterial co-colonizing flora. For example, *Pseusomonas aeruginosa*, known to coexist in the cystic fibrosis lungs with *C. albicans*, has been demonstrated to affect yeast to hyphal transition and also biofilm formation, potentially limiting *C. albicans* to a commensal state of growth (Morales et al., 2013). In contrast to non-oral mucosal sites, the oropharynx has been demonstrated to be more resistant to *Candida* overgrowth than the lower GI tract and vagina following antibiotics (Maraki et al., 2003; Kim et al., 2014). It is not clear, however, if this is due to lack of profound perturbation of the oral bacteriome following antibiotic intake, or perhaps because of less dependency between fungi and bacteria in the mouth. Longitudinal human evidence is thus required on possible changes in global bacterial and fungal profiles during development of oral candidiasis. Similarly, although recent *in vitro* and animal models have suggested a possible role for *Candida-Streptococcus mutans* synergism in the pathogenesis of caries (Falsetta et al., 2014), longitudinal human studies are needed to evaluate the bacterial and fungal microbiome components simultaneously during the progression of this disease.

## Going back to the bench to dissect mechanisms mediating relationships in model systems

Although co-occurrence and co-exclusion patterns inferred from microbial profiles may be used to generate hypotheses, it should be noted that these associations could simply indicate microorganisms with similar nutritional requirements or niche preferences and may not represent direct mutualism or antagonism. Ultimately, the consequences of fungal-bacterial interactions identified clinically require testing in relevant model systems. Our group has used several *in vitro* and animal models to explore the consequences of a fungal-bacterial interaction likely to occur in the human oral cavity.

The genus *Streptococcus* is highly abundant at oral sites, with Mitis group streptococci (MGS) being the most numerically dominant (Dewhirst et al., 2010; Diaz et al., 2012a). MGS, principally represented by *Streptococcus gordonii, Streptococcus oralis, Streptococcus sanguinis*, and *Streptococcus mitis* colonize both teeth and oral mucosal surfaces (Frandsen et al., 1991; Diaz et al., 2006, 2012a). An overgrowth of *Candida* on mucosal surfaces is associated with the appearance of white detachable lesions commonly known as oral thrush (Lalla et al., 2013). Although no evidence from human longitudinal studies of oral candidiasis is available, several lines of evidence point to MGS as potential partners for *Candida albicans*, the most common *Candida* species associated with thrush. *S. oralis* and *C. albicans* are frequently co-isolated from the sputum of antibiotics-treated symptomatic cystic fibrosis patients (Maeda et al., 2011). Furthermore, *C. albicans* and *Streptococcus pneumoniae*, a non-oral MGS with a high degree of genetic and phenotypic relatedness to *S. oralis* (Johnston et al., 2010; Denapaite et al., 2012) have been implicated in pulmonary infections (Yokoyama et al., 2011). Recent evidence has also shown that introduction of *C. albicans* in the intestinal mucosa of antibiotics-treated mice leads to a preferential re-colonization by enterococci (Mason et al., 2012a) and streptococci (Filler, personal communication). Characterization of the cellular composition of thrush lesions in humans is not available, and therefore animal models have been used to investigate the pathophysiology of this condition. Using a murine model of oropharyngeal candidiasis, we showed that thrush-like lesions are formed by densely packed *Candida* cells surrounded by indigenous murine bacterial cocci (Dongari-Bagtzoglou et al., 2009). Furthermore, microscopic studies have revealed corn-cob-like structures formed by *Candida* and streptococci in the mouth of humans (Zijnge et al., 2010). *In vitro* studies have also shown that *Candida* and streptococci physically interact via specific adhesin-receptor mediated binding forming biofilm structures on abiotic surfaces (Silverman et al., 2010). Collectively this evidence supports the idea that *Candida* and streptococci may form a potentially mutualistic partnership.

To begin to study interactions between *C. albicans* and MGS members in models relevant to oral disease we developed an *in vitro* organotypic mucosal model that incorporates salivary flow and also developed an oral polymicrobial infection mouse model. Using these models we showed that when *C. albicans* is co-inoculated with *S. oralis* on mucosal surfaces, streptococcal mucosal biofilm formation is enhanced (Diaz et al., 2012b; Xu et al., 2013). The next logical question to investigate was the role of streptococci in the progression of oropharyngeal candidiasis.

Despite the fact that oral MGS have been traditionally considered avirulent commensals, recent experimental evidence is unraveling a more “sinister” role for these cocci as accessories to primary pathogens in mucosal infections. Using a mouse model of oral infection we recently provided evidence for the role of MGS as accessory pathogens in oropharyngeal candidiasis (Xu et al., 2013). Two MGS species were tested (*S. oralis* and *S. gordonii*) and neither showed virulence on their own, even when animals were immunocompromised and inoculated with a high number of organisms. However, when co-inoculated with *C. albicans, S. oralis* (but not *S. gordonii*) triggered increased frequency and severity of oral lesions, and greater weight loss. Oral co-inoculation with both organisms also triggered an exaggerated mucosal inflammatory response. The majority of the immune regulatory genes upregulated in co-infected animals belonged to the categories of chemotaxis response, neutrophilic response, cytokine activity, and phagocytosis. Interestingly, strong induction of multiple neutrophil-activating cytokines (IL-17C, CXCL1, MIP-2/CXCL2, TNF, IL1α, IL-1β) with concomitant increased neutrophilic infiltration was observed in co-infected animals. Because increased *S. oralis* mucosal colonization in the presence of *C. albicans* aggravated mucosal infection, it is conceivable that like many opportunistic pathogens, a critical mass of this species, reached only in the presence of *Candida*, is needed to induce pathology. These results dispute the long held belief that the commensal bacterial flora protect the host against oral candidiasis (Liljemark and Gibbons, 1973).

*In vitro* biofilm models and rodents have also been recently used to study the interaction of *C. albicans* and *Streptococcus mutans* in the context of dental caries (Gregoire et al., 2011; Falsetta et al., 2014) As seen with MGS, *S. mutans* biofilm formation is enhanced in the presence of *C. albicans*. This interaction appears to be mediated by extracellular polysaccharides. Using a rat caries model, this group also demonstrated that co-inoculation of *C. albicans* and *S. mutans* increased the oral infection burden for both organisms and produced more severe caries lesions than in mono-infected animals (Falsetta et al., 2014).

Evidence on the potential synergy between *Candida* and streptococci from experimental models highlights the need to understand fungal bacterial relationships in the context of human disease. Although human evidence confirming the role of fungal-bacterial interactions in the progression of oropharynegal candidiasis and caries are still required, it is clear that such fungal-bacterial relationships should be considered as potential preventive and therapeutic targets.

## Conclusions

A microbial community has emergent properties, that is, community characteristics cannot be inferred by studying its components separately. In this respect the advent of “omics” approaches will facilitate understanding of microbial communities in humans, using systems biology approaches. We argue, however, that such “omics” methods need to be coupled with adequate *in vitro* and *in vivo* models allowing mechanistic proof for interactions and relationships observed *in vivo* (see Figure 1). Laboratory models can also be used to develop interventional strategies to interfere with fungal-bacterial interactions important in disease development. The development of more complex polymicrobial laboratory models, however, is desirable. Ultimately, proof that interference with fungal-bacterial interactions could serve as an interventional tool for disease needs to be obtained in the complex human ecosystem.

### Conflict of interest statement

The authors declare that the research was conducted in the absence of any commercial or financial relationships that could be construed as a potential conflict of interest.
